# Vaccination and Antiviral Treatment against Avian Influenza H5Nx Viruses: A Harbinger of Virus Control or Evolution

**DOI:** 10.3390/vaccines11111628

**Published:** 2023-10-24

**Authors:** Ahlam Alasiri, Raya Soltane, Akram Hegazy, Ahmed Magdy Khalil, Sara H. Mahmoud, Ahmed A. Khalil, Luis Martinez-Sobrido, Ahmed Mostafa

**Affiliations:** 1 Department of Basic Sciences, Adham University College, Umm Al-Qura University, Makkah 21955, Saudi Arabia; ajasiri@uqu.edu.sa (A.A.); rasoltan@uqu.edu.sa (R.S.); 2 Department of Agricultural Microbiology, Faculty of Agriculture, Cairo University, Giza District, Giza 12613, Egypt; akram.hegazy@agr.cu.edu.eg; 3 Texas Biomedical Research Institute, San Antonio, TX 78227, USA; akhalil@txbiomed.org; 4 Department of Zoonotic Diseases, Faculty of Veterinary Medicine, Zagazig University, Zagazig 44519, Egypt; 5 Center of Scientific Excellence for Influenza Viruses, National Research Center, Giza 12622, Egypt; su.hussein@nrc.sci.eg; 6 Veterinary Sera and Vaccines Research Institute (VSVRI), Agriculture Research Center (ARC), Cairo 11435, Egypt; ahme_2001@hotmail.com

**Keywords:** vaccination, antiviral treatment, avian influenza viruses, H5Nx, virus evolution

## Abstract

Despite the panzootic nature of emergent highly pathogenic avian influenza H5Nx viruses in wild migratory birds and domestic poultry, only a limited number of human infections with H5Nx viruses have been identified since its emergence in 1996. Few countries with endemic avian influenza viruses (AIVs) have implemented vaccination as a control strategy, while most of the countries have adopted a culling strategy for the infected flocks. To date, China and Egypt are the two major sites where vaccination has been adopted to control avian influenza H5Nx infections, especially with the widespread circulation of clade 2.3.4.4b H5N1 viruses. This virus is currently circulating among birds and poultry, with occasional spillovers to mammals, including humans. Herein, we will discuss the history of AIVs in Egypt as one of the hotspots for infections and the improper implementation of prophylactic and therapeutic control strategies, leading to continuous flock outbreaks with remarkable virus evolution scenarios. Along with current pre-pandemic preparedness efforts, comprehensive surveillance of H5Nx viruses in wild birds, domestic poultry, and mammals, including humans, in endemic areas is critical to explore the public health risk of the newly emerging immune-evasive or drug-resistant H5Nx variants.

## 1. Introduction

**Figure 1:**
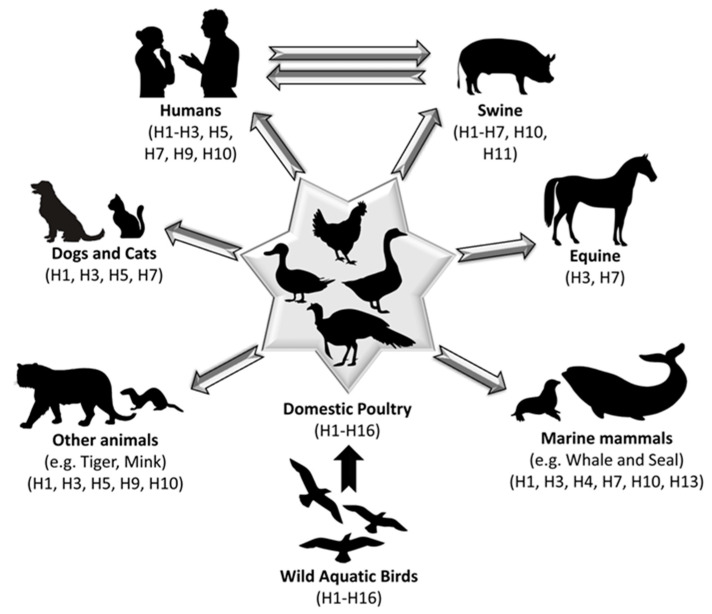
Schematic representation of the disseminated IAV subtypes in different mammalian hosts. Human, swine, marine animal, tiger, mink, horse, and domestic dog and cat AIVs are all assumed to have been transmitted from aquatic wild birds and emerged in avian reservoirs, and domestic poultry, to infect mammalian hosts. Unidirectional arrow refers to zoonotic potential of AIVs while bidirectional arrow refers to potential reverse zoonosis events “human-to-animal transmission” following zoonosis.

Avian influenza viruses (AIVs) belong to the genus influenza A virus (IAV) within the *Orthomyxoviridae* family. The genome of IAV includes eight single-stranded ribonucleic acid (RNA) segments, encoding at least 10 fundamental viral proteins. The viral proteins of IAV can be categorized into (a) surface proteins (hemagglutinin (HA); neuraminidase (NA); and matrix protein 2 (M2); (b) internal structural proteins (polymerase basic 2 (PB2), polymerase basic 1 (PB1) and polymerase acidic (PA); nucleoprotein (NP); matrix protein 1 (M1); and nuclear export protein (NEP)); and (c) non-structural proteins (non-structural protein 1 (NS1)). Currently, 16 HA (H1–H16) and 9 NA (N1–N9) subtypes have been identified as causing AIV infections [1]. In parallel, some bat-origin IAVs were detected in the New World “American continents” bat species (H17N10 and H18N11 influenza viruses) and in the Old World “Europe, Asia, and Africa” bat species (H9N2-like influenza viruses) [2,3]. Most recently, the spectrum of the AIVs has been extended to include the newly characterized/distinct HA subtype in Common Pochard (*Aythya ferina*), nominally H19 [4]. The natural reservoirs of all AIVs are waterfowl and shore birds from where they can be transmitted to new hosts [1,5] (Figure 1).

IAVs constantly evolve via two main genetic forces. The first is caused by the error-prone viral polymerase genetic mutations that constantly accumulate, leading to structural and functional changes, nominally genetic drift. On the other hand, co-infections of a cell/host by different viruses’ subtypes can result in new virus variants that carry viral genome segments from two or more viruses and often demonstrate novel hybrid viruses of unidentified characteristics in a well-known process, nominally genetic reassortment or antigenic shift [1].

AIVs of all subtypes (H1–H16) exist in their natural reservoirs, wild waterfowl and shorebirds, in a low pathogenic (LP) form and can induce only mild illness in birds, if any, whereas the virulence of certain H5 and H7 AIV strains in birds varies widely, ranging from asymptomatic to highly lethal infections [6]. The pathogenicity of AIVs for domestic poultry was confirmed to be associated with alteration in the proteolytic cleavage site (PCS) of the HA protein from a monobasic (e.g., PQIETR▼GLF) to a polybasic amino acid motif (e.g., PQRERRRKKR▼GLF). This alteration facilitates the HA cleavability of highly pathogenic (HP) AIVs by ubiquitous furin-like host proteases, not only in the respiratory and digestive tracts but also throughout the host vital systems, inducing a systemic infection. Besides the PCS, it is also important to note that the amino acid (aa) residues in its vicinity are also important as replication and virulence determinants in mammals [7]. Moreover, virulence markers were documented in structural and non-structural influenza proteins which extend beyond the HA [8,9,10,11]. Thus, the virulence of AIVs is a multigenic trait, requiring a specific genome constellation for full virulence [12,13].

In the last two decades, zoonotic infections of humans caused by LP or high-pathogenic (HP) AIVs have dramatically increased [1,14]. Transmission of the H5N1, H5N6, H7N3, H7N7, H7N9, H9N2, and H10N8 subtype AIVs from poultry to humans has been reported to the World Health Organization (WHO) [1]. Since 2003, 457 out of 868 confirmed human laboratory cases died due to infections with H5N1 [15]. Moreover, 515 out of 1568 cases died due to infections with H7N9 [1]. For H9N2, a total of 89 cases of human infections, including two deaths, were reported from 2015 to 2023 by the WHO in the Western Pacific Region, mostly in China [16]. The rate of infection due to subclinical or undetected cases is thought to be considerably more than currently documented cases. Consequently, the transmission of AIVs to humans poses a serious pandemic risk.

## 2. Introductions and Evolutionary Events of AIVs in Egypt

**Figure 2:**
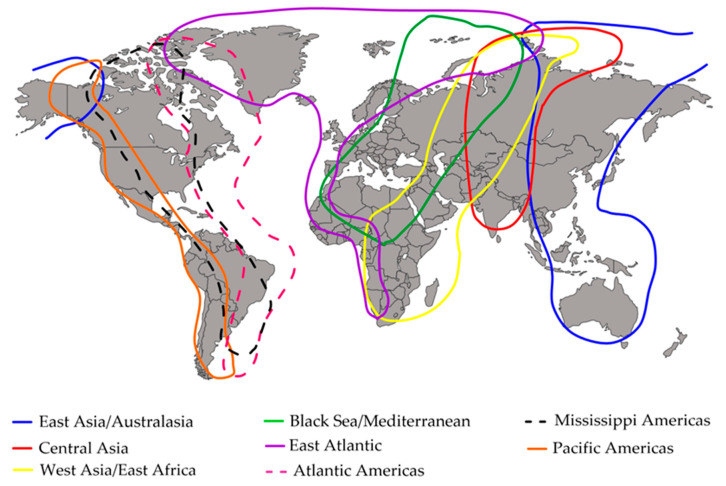
The migration routes (flyways) of migratory birds all over the world. The eight flyways include one main flyway that passes by Egypt and connects west Asia with east Africa. Global flyway boundaries in this map were created according to Boere and Stroud, 2006 [19].

Egypt is a habitat for an impressive number (378) of bird species moving through 34 important bird areas (IBAs) [17] via two major migratory flyways: the Black Sea–Mediterranean and West Asia–East Africa flyways. Both flyways overlap in Egypt at the Nile Delta and the North Mediterranean Coast of Egypt, respectively, providing stopover areas for millions of migratory birds [18]. Moreover, Egypt is particularly important for migratory soaring birds (MSBs), which depend on hot thermal uplifts to fly over and cross narrow water bodies without much effort, such as storks, ibises, pelicans, cranes, and many birds of prey. The Rift Valley/Red Sea Flyway is one of the main flyways for migratory soaring birds in the world. Egypt is of critical passage for these birds as it is located on the only land bridge between the Eurasian and African landmasses that links breeding grounds in Europe and Asia with wintering areas in Africa. The area of Southern Sinai and the Gulf of Suez are very critical to MSB migration as they include some bottlenecks of migration. On the bottlenecks, the flyway divides into two main corridors of migration along the river Nile and the Red Sea Cost (Figure 2).

In 1996/1997, H5N1 AIVs with the potential to infect humans were first isolated in birds from live bird markets (LBMs) in Hong Kong. The parent H5N1 AIVs were isolated from geese in Guangdong province, China, (Gs/Gd) in 1996 and they acquired gene segments from H9N2, H6N2, and H5N1 AIVs. This Gs/Gd AIV killed 6 out of 18 human cases in Hong Kong [20]. Effective control measures and closure of LBMs successfully eradicated the virus. However, the precursors circulating in the wild bird reservoir resulted in the re-emergence of the virus in 2002/2003. In 2005/2006, this virus was transmitted mainly by wild birds to about 60 countries in Asia, Europe, and Africa, causing severe socioeconomic losses in the poultry industry, particularly in developing countries. The virus became endemic in poultry in several countries, including China, Indonesia, Thailand, Vietnam, and Egypt. Phylogenetically, the H5-type HA was diversified into ten clades (clades 0 to 9) and several second-, third-, fourth-, or fifth-order clades (e.g., clades 2, 2.3, 2.3.4, 2.3.4.4, 2.3.4.4b) [21].

**Figure 3:**
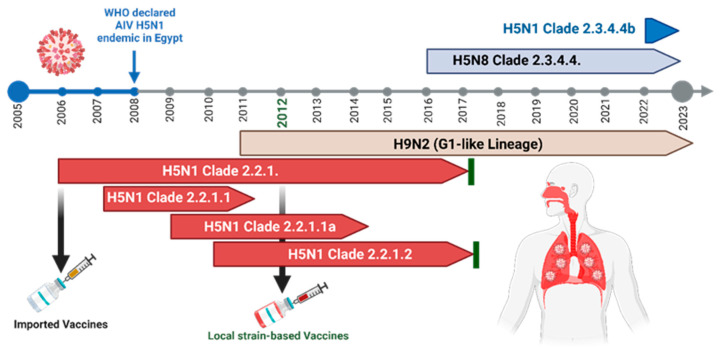
Timeline of different avian influenza viruses, including H5Nx viruses that emerged in the Egyptian poultry sector. From 2006 to 2017, the HPAI H5N1 virus of clade 2.2.1 and its subclades 2.2.1.1 and 2.2.1.2 resulted in devastating economic losses in poultry and a remarkable public health hazard with zoonotic potential in humans. From 2016 to the present, the HPAI H5N8 virus of subclade 2.3.4.4 has been circulating, resulting mainly in losses in poultry. Recently, in 2022, HPAI H5N1 reassortants of clade 2.3.4.4b have been documented in migratory birds and domestic poultry in Egypt.

In Egypt, the HPAI H5N1 was documented in domestic poultry in early 2006 (Figure 3) shortly after its detection in wild migratory birds in Damietta Governorate in late 2005 [22]. This virus continued to circulate, leading to accumulated amino acid substitutions in the surface immunogenic glycoproteins (HA and NA), and the virus was declared as endemic in Egypt in 2008 [17,23]. From late 2009 to 2011, two vaccine-escape H5N1 mutant subclades, namely 2.2.1 and 2.2.1.1, co-circulated and were detected in poultry [17]. Meanwhile, the H5N1 viruses of subclades 2.2.1 and 2.2.1.1 continued to change under improper vaccination circumstances to form new phylogenetic clusters, namely 2.2.1.2 and 2.2.1.2a [17,24,25].

Synchronously, several clades of the LP influenza A/H9N2 viruses (e.g., G1, Y280, and Korean lineages) are well entrenched or endemic in several countries in Asia, Europe, and the Middle East, representing the most widespread AIV subtype in birds worldwide [26]. H9N2 infections in poultry are mostly mild, with slight respiratory symptoms and a sudden drop in egg production (14–75% in breeder or layer flocks) [27]. In 2011, H9N2 AIVs were first detected in Egypt in a quail farm [28]. Although H9N2 viruses are LP in poultry, they pose a serious public health threat. The H9N2 AIVs of the G1-like lineage were isolated from humans in China, Hong Kong [29,30], and Egypt [31]. In early 2015, three infections with H9N2 AIVs were documented among Egyptians that were recently exposed to infected poultry, confirming the high zoonotic potential of H9N2 viruses and ranking Egypt in the third position after China and Bangladesh in the cumulative numbers of H9N2 AIV human infections [32,33]. Importantly, in China, H9N2 viruses donated internal-protein coding gene segments to other AIVs that then became fatal in humans, like the parent H5N1 Gs/Gd in 1996/1997 [34], H10N8 in 2013 [35,36], H7N9 (circulating since 2013) [34,37], and H5N6 in 2014 [38].

In 2013/2014, H5N8 viruses of clade 2.3.4.4 spread from China to Korea and Japan, and then to Siberia, Europe, and Beringia and thereafter into the United States (US) via wild migratory birds [39,40]. This is currently the most widespread H5-type AIV in recent history. The parent viruses from 2014/2015 were characterized by a high mean chicken infectious dose, lack of seroconversion in surviving chickens, low replication in different organs, a transmissibility indicating poor adaptation to poultry, and were clearly less virulent than the parent Gs/Gd virus [41]. In ducks and geese, H5N8 viruses replicated and were excreted to a certain extent without inducing clinical signs or mortality, representing high adaptation [41,42]. Moreover, a hallmark of the 2014/2015 outbreak in the US and elsewhere was the poor adaptation to mammals (mice, ferrets, dogs, cats), demonstrating mild or absent clinical signs and lack of transmission among experimental animals [43,44]. Several studies showed that serial passages in mice increased virulence and viral replication in mammals, which was accompanied by mutations in the PB2, PB1, PA, HA, and/or NP segments [45]. The virus had a lower affinity to human-type receptors, replicated at lower levels than human H1N1 and H5N1 viruses in human cell cultures, and was highly susceptible to neuraminidase inhibitors (NAIs) (e.g., oseltamivir, zanamivir, peramivir) [43,46].

In 2016, another wave of H5N8 AIVs (designated hereafter as H5N8-2016) occurred worldwide [47]. In Europe, H5N8 infections caused severe mortality in wild and domestic birds within a few months in many European countries [48,49,50,51,52]. Five main features of this second wave of H5N8 AIVs have been observed: (i) a high flexibility of HA-NA combinations, with several H5 clade 2.3.4.4 and N1, N2, N3, N5, N6, and N8 combinations [53]; (ii) a high genetic diversity within H5N8-2016 viruses; (iii) multiple introductions in several areas worldwide, indicating higher fitness and endemicity in wild bird reservoirs [49,50,51,54]; (iv) increased pathogenicity in domestic ducks and wild birds compared to H5N8-2014 infections, but low zoonotic potential [49,51,55]; and (v) partially reported airborne transmission between poultry premises [52].

## 3. Imported and Locally Produced Avian Influenza Vaccines in Egypt

In an attempt to alleviate the risk of avian influenza (AI) infections in domestic poultry, vaccination has been adopted as a control strategy in South East Asia since 2003 and in Egypt since 2006 [56]. In Egypt, several avian influenza vaccines engineered from both non-matching and matching strains were imported and subjected to evaluation and approval for field immunization (Table 1). The main criteria of an effective AI vaccination are: (1) vaccine composition that matches the circulating field strains; (2) reliable cold chain and logistics to target all poultry farms, including smallholder poultry farms; and (3) availability of sufficient financial and human resources to ensure the validity of the vaccination campaigns [57]. An improper choice of vaccine strain or the incorrect implementation of vaccination campaigns can also contribute to the emergence of new field variants [58].

To approve the use of imported or locally generated AI vaccines in Egypt, an experimental assessment of the vaccine efficacy must be performed. The efficacy of a vaccine in controlling laboratory challenge infection with a highly pathogenic circulating virus in vaccinated poultry must exceed 80%, with a remarkable drop in virus shedding in vaccinated poultry when compared to the unvaccinated control group [59]. Nevertheless, the effectiveness of an approved vaccine in the field remains unquantified and may differ from the experimental evaluation of “efficacy” due to many factors, including poor biosecurity, timing of vaccination, improper application, the circulation of different quasispecies or subclades of the same virus, and coinfections in poultry flocks that may impact the overall efficacy [57,60]. Therefore, the adoption of laboratory vaccine efficacy as a sole criterion to deduce vaccine field effectiveness is sometimes deceptive.

As illustrated in Table 1, different H5-type viruses were used as vaccine seed strains, including non-circulating clades (e.g., clades 0, 1, and 2.3.2) and lineages (e.g., classical, Eurasian, and north American H5-lineage viruses). Those partially matching vaccine strains showed variable reactivity against earlier antigens and were approved for application, but reactivity declined over time with virus evolution due to antibody-mediated evolutionary selection pressure [61].

**Table 1:** List of some vaccines implemented against AIVs in Egypt [61,62,63].

AIV Subtype	Vaccine Name/ Company/Country	Vaccine Type	Donor Strain	Clade/Lineage	Efficacy *
H5N9	Optimune AIV/Ceva/Mexico	Inactivated	Influenza A/turkey/Wisconsin/1968(H5N9)	Classical H5N1	88%
H5N1/H5N8	ME-Flu VAC/MEVAC/Egypt	Inactivated	Influenza A/chicken/Egypt/RG-173CAL/2017(H5N1); Influenza A/chicken/ME-2018(H5N8)	H5N1 clade 2.2.1.2 and 2.3.4.4b	87–96%
H5N1	SERA-VAC/VSVRI/Egypt	Inactivated	Influenza A/chicken/ Egypt/M2583D/2010(H5N1)	H5N1 clade 2.2.1.1	91–≥99%
H5N1/H5N8	Vallyvac AI H5N1/Valley vaccine/Egypt	Inactivated	Influenza A/chicken/D10552B/2015(H5N1); Influenza A/green-winged tail/Egypt/877/2016(H5N8)	H5N1 clade 2.2.1.2 and 2.3.4.4b	87–≥99%
H5N1	Egy flu/Harbin Veterinary Research Institute/China	Inactivated	Influenza A/chicken/Egypt/18-H/2009(H5N1)	H5N1 clade 2.2.1.1	89–94%
H5N1	Avian Influenza H5 Re6 + Re8 Vaccine/Harbin/China	Inactivated	Influenza A/duck/Guangdong/S1322/2006(H5N1); Influenza A/chicken/Guizhou/4/13(H5N1)	H5N1 clade 2.3.2. and 2.3.4.4b	95–97%
H5N2	Nobilis Influenza H5N2/Intervet/US	Inactivated	Influenza A/duck/Potsdam/1402/86(H5N2)	H5N2 Eurasian	87–92%
H5N2	CEVac Flukem/Ceva/Mexico	Inactivated	Influenza A/Chicken /Mexico/232/94/CPA(H5N2)	H5N2 North American	75–90%
H5N3	Zoetis H5N3/Zoetis/US	Inactivated	Influenza A/chicken/Vietnam/C58/2004(H5N3)	H5N1 clade 1	90–97%
H5N1	Volvac^®^ B.E.S.T. AI + ND)/Boehringer Ingelheim/Mexico	Inactivated	Influenza A/duck/ China/E319-2/2003(H5N1)	H5N1 clade 2.3.2	93–98%
H5N1	Reassortant AIV (Subtype H5N1) Vaccine (strain Re-1)/Zhaoqing DaHuaNong Biology Medicine, Sihui, China	Inactivated	Influenza A/goose/Guangdong/1996(H5N1) (Re-1)	H5N1 clade 0	94%
H5N1	Reassortant AIV (strain Re-5) Re-5/Merial (USA) and QYH (China)	Inactivated	Influenza A/duck/Anhui/1/2006(H5N1) (Re-5)	H5N1 clade 2.3.4	93–98%
H5N1	Egymune/Yebio Bioengineering company/China	Inactivated	Influenza A/duck/Anhui/1/2006(H5N1) (Re-5)	H5N1 clade 2.4.4.	95%
H5N1	Reassortant Re-8/Merial (USA) and QYH (China)	inactivated	Influenza A/chicken/Guizhou/4/2013(H5N1) (Re8)	H5N1 clade 2.3.4.4	95–97%

* Vaccine efficacy was evaluated by central governmental and research laboratories against different emerging subtypes/clades.

## 4. Improper Antiviral Drug Prescription to Control AIVs in Avian Species and Its Impact on the Evolution of Drug-Resistant Variants

In addition to the application of vaccines to control IAV infections, antivirals are considered a major line of defense with therapeutic activities against IAVs [64]. Three classes of IAV antivirals were approved for the treatment of human influenza virus infections: M2 blocker adamantanes (amantadine and rimantadine), neuraminidase inhibitors (NAIs) (oseltamivir, zanamivir, and peramivir), and polymerase acidic (PA) protein inhibitor (Baloxavir marboxil) [65,66,67]. Adamantanes work by blocking the matrix 2 (M2) ion channel protein of the influenza virus that is essential for the fusion of virus and host-cell membranes, thus preventing virus uncoating and the release of vRNPs into the host cell cytoplasm [68]. These M2 blockers have historically been used as a symptomatic treatment for Parkinson’s disease [69]. Due to the extensive use of amantadine in treating influenza virus infections, the percentage of drug-resistant strains increased from 0.4% during 1994–1995 to 12.3% during 2003–2004 [70]. In 2006, the percentage of amantadine-resistant strains continued to increase and 92% of human H3N2 influenza viruses were resistant to amantadine [70]. Therefore, in 2007, amantadine was not recommended by the WHO for the treatment of human virus infections [71].

Although amantadine has not been approved for use in poultry, several reports revealed the widespread illegal use of adamantanes in treating AIV infection in poultry and/or as a nutrient supplement for different poultry species in different countries, including China and Egypt [72,73]. The cheap price of amantadine and lack of strict veterinary supervision facilitated its wide application in the poultry production sector and the subsequent emergence of avian influenza adamantane-resistant strains. Also, the segmented nature of the influenza virus genome and lacking polymerase proofreading ability lead to a high mutation rate of the influenza virus and subsequently its escape from the antiviral effect. Indeed, a single amino acid substitution at positions 26, 27, 30, 31, and 34 in the transmembrane region of M2 is sufficient to confer virus resistance to amantadine [74]. Hence, several studies reported the emergence of amantadine-resistant AIVs worldwide [75,76]. In Egypt, AIV amantadine-resistant mutants emerged in 2007 among H5N1 isolates of different clades, harboring mainly the amino acid substitutions V27A and S31N, then continued to circulate until 2011 [77,78]. Amantadine-resistant mutants were not detected before the re-emergence of H5N1 IAVs in 2015 [77,79]. In addition to H5N1 AIVs, amantadine-resistant mutants were reported in Egypt among H9N2 AIVs strains [80], which are currently endemic in Egypt and co-circulating with H5N1 and other AIV subtypes in poultry species with zoonotic potential to induce human infections [28,81,82].

Due to the unavailability of rimantadine, the second M2 blocker, in most countries, there are no reports of using it in poultry. Nevertheless, H7N9 AIVs isolated in China, which induced several human infections, were resistant to both rimantadine and amantadine [83].

Oseltamivir is one of the most used NAIs and is stockpiled as a part of pandemic preparedness plans [84]. Oseltamivir interacts with the virus’s NA and hinders its function in mediating the cell release of new virions from infected cells [85]. While oseltamivir is currently the drug of choice for the treatment of influenza infections, oseltamivir-resistant mutants have emerged among different influenza subtypes. However, due to the structural difference in the NA of different influenza subtypes, mutations responsible for conferring oseltamivir resistance are also different: amino acid substitutions H274Y (N2 numbering) for A/H1N1 viruses; E119V, R292K, and N294S for A/H3N2 viruses; and R150K and D197N for influenza B viruses [86]. Fortunately, the administration of oseltamivir as prophylaxis in poultry flocks is costly, which renders its wide application difficult; nevertheless, oseltamivir-resistant H5N1 AIVs have been also reported [87,88,89,90]. Experimentally, several studies revealed the development of oseltamivir-resistant mutants in aquatic birds (mallards) after exposure to oseltamivir treatment and the ability of these mutants to transmit to domestic chickens [91,92]. Although oseltamivir-resistant IAVs are documented in humans [93,94], the drug is still effective and is considered the drug of choice in treating human influenza infections. However, the application of oseltamivir as a prophylactic or therapeutic against AIV infections in birds will accelerate the emergence and widespread of oseltamivir-resistant mutants that subsequently will limit the treatment options for human influenza infections.

Another NAI is zanamivir, which also works as a synthetic analog of sialic acid receptors to hinder influenza virus infection. While oseltamivir is the drug of choice in treating human influenza virus, zanamivir is often used when the effect of oseltamivir is limited. For influenza A/H1N1 viruses, the amino acid substitution H274Y is proved to confer oseltamivir resistance while I223R substitution could reduce the effectiveness of both oseltamivir and zanamivir [95]. Most of the zanamivir resistance incidences were reported after a period of treatment in immunocompromised patients [96,97,98].

Baloxavir marboxil (BxM) was developed as a new anti-influenza drug via inhibiting the cap-dependent endonuclease activity of the polymerase acidic (PA) protein which is essential for the generation of capped RNA primers (mature mRNA) for viral transcription [99]. BxM was first approved in Japan and the US in 2018, and since then it has been used in several other countries. Amino acid substitutions at position 38 of PA correlated with decreased effectiveness of BxM [100]; however, the degree of resistance varies according to the substituted amino acid. Briefly, leucine (L) confers a 10-fold and a 3-fold reduction in the susceptibility of A/H1N1 and A/H3N2 IAVs, respectively [99,101,102]. Also, the amino acid substitutions threonine (T), methionine (M), or phenylalanine (F) confer a 10- > 50-fold reduction in the susceptibility to BxM, while valine (V) does not seem to have any effect [102]. Virological surveillance conducted in Japan during the 2018–2019 influenza season revealed that reduced susceptibility to BxM has been reported at a low frequency, but did not spread widely during the 2019–2020 influenza season due to amino acid substitutions at position 38 of the PA (I38T and 38F) [103].

Overall, antivirals available for the treatment of human influenza virus infections are limited, particularly with the rapid emergence of antiviral-resistant variants. However, the application of any of these antivirals to control AIV infection in birds will undoubtedly increase the prevalence of antiviral-resistant variants, which will thereby result in reduced susceptibility or loss of activity of these antivirals in humans.

## 5. Adaptive Mammalian Mutation Markers in Egyptian AIVs

AIV circulation in animal reservoirs under subclinical control measures results in the emergence of genetic traits which permit the virus to cross the avian-to-human species barrier. These acquired genetic changes to improve the viral fitness of zoonotic AIVs can be gained either in avian hosts or the new mammalian host [1]. However, we assume that the minimum essential genetic changes to initiate the zoonosis process are likely to occur in the original avian host before transmitting to mammals, including humans.

Despite the vaccination programs in poultry in Egypt to control AIV circulation, the majority of the reported human infections with AIV H5N1 worldwide in the last two decades (2003–2023) were documented in Egypt (41%, 359/868) with a considerable case–fatality rate (26%, 120/457) [15]. We assume that these high morbidity and mortality rates might be a potential consequence of prophylactic antiviral drug misuse for poultry and/or the application of non-matching/partially matching vaccine strains to control the circulating and emerging AIVs.

A diverse range of AIVs have infected various mammalian hosts during the past two decades and have posed a persistent threat to both human and animal health. This is an outcome of breaking down the interspecies barrier, which is primarily attributed to non-silent changes in the viral genome. The molecular basis for interspecies transmission of influenza viruses involves several factors that affect the transmissibility of these viruses between avian and mammalian host species. Several molecular characteristics are crucial for interspecies transmission, including (1) HA receptor binding specificity; (2) HA fusion stability; (3) the presence or absence of potential N-glycosylation sites in the HA; (4) the increased viral replication by the viral ribonucleoprotein (vRNP) complex in a new host; (5) the balance between receptor binding and fusion via HA protein and the receptor-cleaving capacity of the NA protein in the new host; and (6) genetic reassortments that result in different gene constellations [104,105,106]. The tripartite polymerase complex (PB2, PB1, and PA), together with viral HA, control the ability of influenza viruses to infect different species [107,108]. As the binding preferences of HA in avian and human influenza viruses are α2,3-SA and α2,6-SA, respectively, it is believed that sialic acid (SA) receptors act as a host barrier in AIV zoonotic potential and interspecies transmission. For interspecies transmission, an alteration of the binding preference is needed so that the HA of AIV can bind the mammalian- or human-type SA receptor (α2,6-SA) [108,109,110]. Furthermore, the viral polymerase complex has a crucial role in viral adaptation in the new hosts and is a major determinant of pathogenicity because it is considered the cornerstone of viral genome replication and gene transcription [111,112,113,114,115,116]. Mainly, this adaptation to the new host is caused by single or multiple mutations in the genome of avian and human influenza viruses. A positive selection of certain amino acid substitutions, encoded by single or multiple mutations in the viral genome following virus adaptation to the new host, is considered an adaptive mutation marker. Interestingly, multiple sporadic human illnesses have been reported following adaptive mutations in the PB2 polymerase subunit [117,118,119,120]. PB2-E627K substitution is the most commonly occurring human adaptation polymerase mutation [121,122,123]. The high virulence of H5N1 AIV in mice and the ability to replicate effectively in the mouse’s upper respiratory tract (URT) were shown to be associated with the presence of E627K adaptive mutation in the PB2 gene [124,125,126]. Moreover, the replication of AIV in mammalian cells was boosted by the human virus-like residue T271A in PB2 [127]. Additionally, it has been shown that certain mutations in PB1, such as L473V and L598P, increase AIV polymerase activity in mammalian hosts [128,129]. Researchers have also investigated the function of other viral proteins, including HA, NS1, NEP, and matrix proteins M1 and M2, in mammalian transmission [130,131,132,133,134,135,136].

Because of its distinct characteristics, the HPAI H5N1 currently imposes serious impacts on both animal and human health. Even though H5N1 viruses have been implicated in hundreds of human cases, this subtype lacks the potential for prolonged transmission in humans, which may be owing to the avian-type receptor-binding specificity in HA (α2,3-SA) [137,138]. Molecular factors, particularly changes in HA that affect its receptor-binding specificity by either enhancing the binding to α2,6-SA or reducing the binding affinity to α2,3-SA, have been linked to multiple sporadic infections in humans [137,139]. Numerous amino acid substitutions in HA, including Q226L, G228S, R193K, and E190D, have been found to improve the binding of HPAI H5N1 HA to α2,6-SA in both natural and experimental settings [137,138,139,140,141,142,143]. Three other mutations were also identified as being crucial for the respiratory droplet infectiousness of H5N1 virus in ferrets, including H99Y in PB1 and H110Y and T160A in HA [113]. It has been shown that an airborne-transmissible H5N1 might be produced by N224K and Q226L mutations together with the HA substitutions N158D and T318I, which change the receptor binding preference [144]. As seen in H5N1 with the T160A amino acid change, the deletion of the glycosylation site close to the receptor-binding domain (RBD) at position 158–160 is favorable for human receptor specificity, increasing the binding to human-type receptors [113,144,145]. Furthermore, it was shown that the amino acid substitutions L473V and L598P in PB1 increased the H5N1 virus’s polymerase activity in human cells and enhanced the replication of the pandemic H1N1 virus [129].

Despite the fact that no human infections with H5N8 AIVs have been reported since its emergence in 2014, a genomic investigation of H5N8, which belongs to clade 2.3.4.4, revealed several human-like mutation markers, including PA-404S, PB2-613I, PB2-702R, HA-137A, HA-227R, and HA-A160T [145,146]. The A160T amino acid substitution, which is an important glycosylation site in the HA protein, could potentially increase binding specificity for human-type receptors and increase the transmissibility of H5N8 AIVs from avian species to mammalian models [147].

Regarding H9N2 viruses, it was found that L226Q in HA improved the efficiency of direct transmissibility, and it was also shown that a higher incidence of the L226Q-containing H9N2 viruses in China increased the risk of human infection with this subtype [148]. Swine H9N2 viral isolates were found to have distinctive amino acid alterations in the HA at residue 227 inside the receptor-binding pocket and residues 274, 279, and 286 outside the receptor-binding area [149]. Moreover, the affinity for the α2,6-SA human receptor has also been found to be influenced by position 200, with valine (V) exerting the highest affinity, threonine (T) showing intermediate affinity, and alanine (A) demonstrating the lowest binding affinity [150]. Moreover, H9N2 viruses were experimentally able to infect and disseminate in ferrets via respiratory droplets by promptly acquiring typical mammalian-adaptive markers including E627K, D701N, and Q591K mutations in PB2 with or without adaptation to mammalian hosts [151,152].

A cumulative summary of the distinctive molecular markers among currently circulating AIVs in Egyptian poultry is illustrated in Table 2. Interestingly, some of these adaptive mutations were acquired in poultry before transmission to mammals or humans, representing the minimum essential elements to recognize mammalian or human host cells and infect them. Following transmission to humans or mammals, the virus acquires several host-specific adaptive mutations due to differences between its original natural hosts and the mammalian hosts, including humans [1]. Table 3 summarizes the impact of certain adaptive amino acid mutations/substitutions on the characteristics of AIVs in mammalian systems.

## 6. Conclusions and Perspectives

Despite the fact that the risk of H5Nx virus transmission to the public is still low, close monitoring of these AIVs and persons exposed to them is imperative [275]. These AIVs are continuously evolving in endemic areas with improper control plans in place, and under inadequate immune and drug pressures. Taking into consideration the COVID-19 scenarios and the evolution of immunoescape variants in certain geographical areas, followed by the devastating spatiotemporal transmission of these SARS-CoV-2 variants of concern (VoCs) in a few days/months to all continents, we urge global health systems within the “One Health” approach to detect signals of potential variants of interest (VOIs) or variants of concern (VOCs) for the newly emerging avian influenza H5Nx viruses and rapidly assess their risk(s). Likewise, the application of evolution-driving control strategies, including vaccination, in certain geographical areas of the world must be subjected to assertive regulations, including safe farming practices and implementation of locally matching vaccine strains, because these viruses can affect the country of origin, neighboring countries, and may pave the way for a devastating pandemic if a virus acquires the minimum essential substitutions that support viral infection and person-to-person airborne transmission. Therefore, international collaboration to implement unified control strategies against AIVs must be urgently established and applied properly among the different sectors of the One Health approach.

## Figures and Tables

**Table 2:** Documented avian-to-mammalian adaptive mutations in AIVs in Egypt.

Viral Protein	Residue	AIV H5N1			AIV H5N8			AIV H9N2		
	aa Position	AAMM ^1^	MAMM ^2^	References	AAMM ^1^	MAMM ^2^	References	AAMM ^1^	MAMM ^2^	References
PB2	44	-	-	-	A	S	[153,154,155,156]	A	S	[157]
	63	I	T	[25]	-	-	-	-	-	-
	64	-	-	-	M	T	[153,158]	I/M	T	[157]
	73	K	R	[159]	-	-	-	-	-	-
	81	-	-	-	T	M	[153,156]	T	M	[157]
	89	V	V	[160,161]	-	-	-	-	-	-
	199	A	S	[154,159]	A	S	[153,154,156]	A	S	[157]
	256	D	G	[25]	-	-	-	-	-	-
	309	D	D	[160,161]	-	-	-	-	-	-
	318	-	-	-	-	-	-	K/S/R	R	[157,162]
	339	K	K	[160,161]	-	-	-	-	-	-
	588	A	V	[160,161,163]	-	-	-	-	-	-
	591	Q	K	[25]	Q	K	[153,164]	-	-	-
	627	E	K	[25,121,160,165,166,167,168]	E	K	[153,169]	E/V	K	[157,170]
	661	-	-	-	A/V	T	[153,171]	A	T	[157]
	701	D	N	[25,146,160]	D	N	[153,172]	D	N	[157]
	702	-	-	-	K	R	[153,171]	K	R	[157]
PB1	3	D	V	[173]	-	-	-	-	-	-
	13	-	-	-	L	P	[153,174]	L	P	[157]
	105	N	S	[175]	-	-	-	-	-	-
	207	K	R	[25]	-	-	-	-	-	-
	336	-	-	-	V	I	[153,154]	V	I	[157]
	374	A	S	[165]	-	-	-	-	-	-
	375	-	-	-	N	S	[153,174]	N	S	[157]
	436	Y	H	[25]	-	-	-	-	-	-
	677	T	M	[25,176]	-	-	-	-	-	-
	740	F	L	[165]	-	-	-	-	-	-
PB1-F2	66	S	S	[25,160,177,178]	-	-	-	-	-	-
	68	-	-	-	-	-	-	T/I	I	[157]
	73	-	-	-	-	-	-	K	R	[157]
	76	-	-	-	-	-	-	V	A	[157]
	79	R	Q	[154,159]	-	-	-	R	Q	[157]
	82	L	S	[154,159]	-	-	-	L	S	[157]
PA	28	-	-	-	P	L	[153,179]	P	L	[157]
	55	-	-	-	D	N	[153,154,156]	D	N	[157]
	57	-	-	-	R	Q	[153,154]	R	Q	[157]
	97	T	I	[175]	-	-	-	-	-	-
	100	V/I	A	[25,160,180]	V	A	[153,181]	V	A	[157]
	133	-	-	-	E	G	[153,182]	E	G	[157]
	158	K	R	[165]	-	-	-	-	-	-
	225	-	-	-	S	C	[153,166]	S	C	[157]
	241	-	-	-	C	Y	[153,183]	C	Y	[157]
	268	-	-	-	L	I	[153,166]	L	I	[157]
	312	-	-	-	-	-	-	K	R	[157]
	356	K	R	[25,154]	K	R	[153,154]	K	R	[157]
	382	-	-	-	E	D	[153,156]	E	D	[157,162]
	400	S/T/F	L	[25,156,159]	-	-	-	S	L	[157]
	404	-	-	-	A	S	[153,154]	A	S	[157]
	409	S	N	[25,154,160,180]	S	N	[153,154,156]	S	N	[157]
	515	A/T	T	[160,184]	-	-	-	-	-	-
	552	-	-	-	T	S	[153,166]	T	S	[157]
	556	-	-	-	-	-	-	Q	R	[157]
	615	-	-	-	K	L	[153,185]	K	L	[157]
	712	A	T	[165]	-	-	-	-	-	-
HA	125	-	-	-	-	-	-	A	T	[162]
	155	I	T	[186]	-	-	-	-	-	-
	173	-	-	-	-	-	-	Q	H	[162]
	180	-	-	-	-	-	-	V	E	[162]
	198	E	D	[160,187]	-	-	-	-	-	-
	216	-	-	-	-	-	-	Q/L	L	[162]
	222	-	-	-	Q	L	[153,188]	-	-	-
	224	-	-	-	G	S	[153,188]	-	-	-
	226	-	-	-	-	-	-	Q	L	[157,170]
	228	-	-	-	-	-	-	R	K	[162,170]
	234	Q	L	[160,189]	-	-	-	Q	L	[157,190,191]
	236	G	S	[160,189]	-	-	-	-	-	-
NP	31	-	-	-	-	-	-	R	K	[157]
	33	V/I	I	[25,154,156,159]	V/I/D	I	[153,154,156]	V	I	[157]
	34	-	-	-	-	-	-	D	N	[157]
	61	-	-	-	I	L	[153,156,166]	I	L	[157]
	100	-	-	-	-	-	-	R	V	[157]
	105	M	V	[9,192,193]						
	109	I/V	V	[25,154,156]	I	V	[153,154]	I	V	[157]
	127	-	-	-	-	-	-	E	D	[157]
	136	L	M	[25]	L	M	[153,156]	L	M	[157]
	184	K	K	[11,25,194]	-	-	-	-	-	-
	214	-	-	-	R	K	[153,154,156]	K/N	K	[157]
	283	-	-	-	-	-	-	L	P	[157]
	293	-	-	-	-	-	-	R	K	[157]
	305	-	-	-	-	-	-	R	K	[157]
	313	-	-	-	F	Y	[153,154,156]	F	Y	[157]
	357	-	-	-	Q	K	[153,154]	Q	K	[157]
	372	-	-	-	E	D	[153,154]	E	D	[157]
	375	-	-	-	-	-	-	D	G/E	[157]
	398	-	-	-	K	Q	[153]	K	Q	[157]
	422	-	-	-	-	-	-	R	K	[157]
	434	-	-	-	-	-	-	E	K	[195]
	442	-	-	-	-	-	-	T	A	[157]
	455	-	-	-	D/N/E	E	[153,154]	D/E	E	[157]
M1	15	V	I	[80]	V	I	[153,196]	V	I	[157]
	30	N	D	[197]	-	-	-	-	-	-
	43	I	M	[198]	-	-	-	-	-	-
	115	-	-	-	V	V/I/T	[153,166]	V	I	[157]
	121	-	-	-	T	A	[153,166]	T	A	[157]
	137	-	-	-	T	A	[153,156,166]	T	A	[157]
M2	11	-	-	-	T	I	[153,154]	T	I	[157]
	16	E	G/D	[80]	E/G	G/D	[153,156]	E/G	G/D	[157]
	20	-	-	-	S	N	[153,154,156]	S	N	[157]
	28	I	I/V	[80]	I	I/V	[153,156]	I	I/V	[157]
	55	-	-	-	L	F	[153,199]	L	F	[157]
	57	-	-	-	Y	H	[153,154]	Y	H	[157]
	86	-	-	-	V	A	[153,154]	V	A	[157]
NS1	42	P	S	[25,200]	-	-	-	-	-	-
	55	K	E	[201]	-	-	-	-	-	-
	66	K	E	[201]	-	-	-	-	-	-
	74	D	N	[202]	-	-	-	-	-	-
	92	D	E	[203]	-	-	-	-	-	-
	103	F/L	L	[25,204]	-	-	-	-	-	-
	106	I	M	[205]	-	-	-	-	-	-
	138	C	F	[201]	-	-	-	-	-	-
	149	V	A	[25,160,206,207]	-	-	-	-	-	-
	227	E	K	[80]	G	K/R	[153,199]	E/K	K/R	[80,157,162]
NS2/NEP	16	M	I	[208]	-	-	-	-	-	-
	41	Y	C	[208]	-	-	-	-	-	-
	75	E	G	[208]	-	-	-	-	-	-

^1^ AAMM: avian-adaptive mutation marker;^2^ MAMM: mammalian-adaptive mutation marker.

**Table 3:** Phenotype drift markers in AIVs and their altered characteristics.

Viral Protein	aa Changes	Subtype	Characteristics/Effects of Mutations	References
PB2	I64M	H5N1	- Human host marker.	[209]
	T339M	H5N1	- Enhances polymerase activity and pathogenicity in mice.	[210]
	I147A K339T A588T	H5N1	- Enhance the ability of H5N1 AIV to replicate in mammalian cells.	[211,212]
	E158G T271A	H5N1	- Enhance pathogenicity in mice and viral polymerase activity in mammalian cells.	[212,213,214,215]
	L89V G309D R477G I495V	H5N1	- Compensate the lack of Lys627 or/and contribute to interspecies transmission. - L89V and G309D increase virulence in mice.	[214,216]
	Q591K/R	H1N1 H9N2 H5N1	- Enhances the ability of H1N1, H9N2 and H5N1 viruses to replicate in mammalian cells.	[212,215,217]
	E627K	H5N1 H7N9 H10N8	- Increase transmission, replication, and virulence in mammalian cells and mice. - Facilitate the adaptation of H5N1 AIVs to mammals and increase transmission and pathogenesis in humans.	[35,113,214,215,218,219,220,221]
	D701N S714R	H5N1	- PB2 mutation S714R, in combination with the mammalian signature at position 701, has the potential to promote the adaptation of an H5N1 AIV to a mammalian host. - Mutation D701N led to an increase in polymerase activity and replication efficiency in mammalian cells and in mouse pathogenicity. - This increase was significantly enhanced when mutation D701N was combined with mutation S714R.	[215,222,223]
	S715N	H5N1	- Role in determining the high virulence of H5N1 in mice.	[224]
	E249G G309D T339M	H5N1	- Contribute to an efficient replication of avian H5N1 viruses in human lung A549 cells.	[210,215]
	V108A E192K A274T N456D G727R T339M I451V S471F R369K	H5Nx	- In silico predicted as convergent or parallel evolution sites of H5Nx.	[209,215]
PB1-F2	N66S	H5N1	- Enhances virulence of H5N1 AIV in mice.	
PB1	V3D	H5N1	- Increases PB1 and PA interaction, resulting in decreased replication of pigeon-derived H5N1 viruses.	[212,225]
				[219]
	K363R A374S F740L	H5N1	- Possibly manipulate H5N1 viral replication or host adaptation.	[219]
	H436Y D622G	H5N1	- Increase polymerase activity and virulence in mammalian systems.	[131,184,226,227,228]
	I57T E172D M179I S361G N375S K387R L598P	H5Nx	- In silico predicted as convergent or parallel evolution sites of H5Nx AIVs. - L598P enhances polymerase activity and replication efficiency.	[215]
	P708S	H5N8	- Enhances viral replication and polymerase activity in human cell lines and virulence, including multi-organ dissemination, in mice.	[48]
PA	K158R	H5N1	- Increases polymerase activity in H5N1 AIV isolated from infected pigeons.	[173,219]
	A343S D347E	H5N1	- Increase viral polymerase activity and mouse virulence of H5N1 AIVs, posing an increased risk to humans.	[173,219,229]
	A369V V602I A712T	H5N1	- Possibly manipulate viral replication and/or host adaptation.	[219]
	S224P N383D	H5N1	- Amino acid substitution S224P increases viral replication in duck embryo fibroblasts. - Amino acid substitution N383D increases polymerase activity in duck embryo fibroblasts and delays the accumulation of the PA and PB1 polymerase subunits in the nucleus of virus-infected cells.	[230]
	E31K	H5N1	- Increases viral replication in Vero cells. - Growth determinant of H5N1 in a high-yield AI vaccine backbone.	[231]
	S409N K356R V100A Q/T/S400L	H5N1	- Increase H5N1 adaptation to humans.	[154,232,233]
	T552S T97I K142E I353R T515A P149S R266H L357I T515S	H5N1	- Enhance the growth capability of H5N1 AIVs in mammalian hosts. - K142E enhances polymerase activity in mammalian cells. - I353R contributes to high polymerase activity in mice and changes the innate response. - P149S, R266H, L357I, and T515S have been reported to increase the polymerase activity of H5N1 AIVs in human 293T cells. - T97I enhances polymerase activity and increases virulence in mice.	[184,215,217,234,235,236,237]
	V44I V127A C241Y A343T I573V	H5N1	- Enhance the growth capability of H5N1 viruses in human A549 cells and their pathogenicity in mice.	[215,236]
	R367K	H5N1	- Enables H5N1 viruses to replicate more efficiently in primary and secondary lung-derived cell lines and enhances higher mortality in BALB/c mice.	[238]
	K615R	H5N1	- Increases polymerase activity of H5N1 AIVs. - Increases virulence of H5N1 in mammals. - Host or mammalian adaptation marker.	[215,234]
	A369V	H5N1	- Possibly manipulates viral replication or host adaptation.	[219]
	A37T I38M/T	H5N1 H1N1 H3N2	- Decrease susceptibility to approved PA cap-dependent endonuclease inhibitor (Baloxavir).	[239]
	F4C M12I M86V F105L L226F E237K P257L N321K T369A V387I	H5Nx	- In silico predicted as convergent or parallel evolution sites of H5Nx AIVs. - N321K increases polymerase activity.	[215]
	N409S V63I	H7Nx	- Enhance virulence in mice.	[180,240]
	K356R	H9N2	- Enhances viral polymerase activity, replication efficiency, and virulence in mice.	[241]
HA	H110Y	H5N1	- Increases HA stability and H5N1 virus respiratory droplet transmissibility in ferrets.	[113,212,242]
	S133A T188I	H5N1	- Increase pseudovirus binding to α2,6-SA.	[243,244]
	N158D	H5N1	- Glycosylation site removal in HA and H5N1 virus respiratory droplet transmissibility in ferrets.	[144,212]
	T160A	H5N1	- Glycosylation site removal in HA and H5N1 virus respiratory droplet transmissibility in ferrets.	[113,212]
	Q196R Q226L G228S	H5N1	- Role in receptor-binding specificity and H5N1 virus respiratory droplet transmissibility in ferrets.	[212,245]
	N224K Q226L	H5N1	- Role in receptor-binding specificity and H5N1 virus respiratory droplet transmissibility in ferrets.	[144,212,246]
	Q226L G228S	H5N1	- Role in receptor-binding specificity and H5N1 virus respiratory droplet transmissibility in ferrets.	[113,212]
	T318I	H5N1	- Increases HA stability and H5N1 virus respiratory droplet transmissibility in ferrets.	[144,212]
	K193E G225E	H5N1	- Enhance replication and genetic stability after serial passaging of H5N1 AIVs. - Attenuation of H5N1 virus in mice. - Immunization with these mutations induces robust antibody responses against H5N1 in mice. - K193E and G225E mutations synergistically attenuated H5N1 without enhancing receptor-binding avidity.	[247]
	S221P D95G	H5N1	- S221P affects the heat stability of HA and this effect is enhanced when combined with D95G mutation, which may aid H5N1 virus respiratory droplet transmission in mammals.	[246]
	N186I/T	H5N1	- N186I/T mutations increase virulence in mice. - N186I/T mutations enhance virus replication in the early stages of infection in chicken embryos and increase levels of viral replication at late stages. - N186I/T mutations increase viral replication at lower temperatures.	[248]
	Q222L G224S	H5N1	- Change the receptor binding preference of the HA from avian α2,3-SA to human α 2,6-SA.	[189]
	K193T	H5N1	- Improves both binding to human trachea epithelial cells and specificity for human α-2,6-SA.	[249]
	G186V	H7N9	- A potential adaptation of avian H7N9 viruses to human α-2,6-SA.	[250,251]
	K58I	H5N1	- Associated with increased viral replication in the upper respiratory tracts of mice and ferrets.	[252]
	K58I G219S	H7N9	- K58I, combined with G219S, increases affinity of binding to α-2,3-SA and α-2,6-SA.	[253]
	Q192R/H G222L Q224S	H5N1	- Switch receptor binding specificity from avian to mammalian receptors.	[244,254,255,256]
	Δ129 I151T	H5N1	- Double Δ129/I151T mutations exhibit enhanced binding affinity for human α-2,6-SA while retaining avian α2,3-SA specificity. Increase tropism to the human lower respiratory tract.	[139]
	Δ154–156 T318I H103Y N220K Q222L	H5N1	- Loss of 154–156 HA glycosylation results in enhanced airborne transmission in ferrets. - T318I, H103Y, N220K, and HA-Q222L mutations increase the stability of the HA protein.	[113,144,257]
	S223N K153D G272S	H5N1	- S223N is predicted to increase affinity towards human α-2,6-SA. - S223N with K153D and/or G272S increase affinity of H5N1 viruses to human α-2,6-SA and increased replication in mammalian cell cultures. - S223N is predicted to increase H5N1 virus binding to human α-2,6-SA.	[7,215,258]
	S223I/N Δ129 I151T H125Y N94D A134V N182K T195I	H5N1	- S223N or S223I/Δ129/I151T increase lethality in mice. - H125Y, H125Y/N94D, A134V/S129Δ/I151T, N182K, N182K/T195I, N182K/T195I/N94D, S223N, and S223N(I)/S129Δ/I151T mutations have been shown to increase viral replication in human airway epithelia due to enhanced binding specificity to human α-2,6-SA and increased HA fusogenic activity. - A134V has been included as a virulence marker for H5N1 viruses.	[245,259,260]
	A134V G139R S155N E186G N193K V210I P235S E75K S123P I151T S133A T188I	H5N1	- Increase H5N1 virus binding to human α-2,6-SA.	[215,244,245,261]
	T156A	H5N1	- Increases H5N1 virus binding to human α-2,6-SA and virus transmission in guinea pigs.	[242,262]
	D154N	H5N1	- Increases N5N1 airborne transmissibility in mammals.	[78,215,260,263]
	N182S R497k K189R	H5N1	- Increase H5N1 virus binding to human α-2,6-SA.	[215,244]
	E184G D376N	H5Nx	- Increase H5N1 virulence in mammals.	[215]
	Q30P D31N K35R D45N A86V D88G A127T A/T127S R140K M140T S141P R162I V174I A184E T195A T/N195I V210A V219I R310K R323K R326K I375M D387N E433G N476D E477K M479I E502G M532I V533I	H5Nx	- In silico predicted as convergent or parallel evolution sites of H5Nx viruses.	[215]
NP	K470R	H5N1	- Increases the virulence of H5N1 viruses in mammals.	[264]
	A184K	H5N1	- Increases H5N1 replication and pathogenicity in chickens.	[11]
	N319K	H5N1 H7N7	- Enhances H5N1 and H7N7 viral replication in mammalian cells.	[185,265]
	A284T	H5Nx	- Increases virulence in mice.	[215]
	R100I V343I R384K S413L P419S R452K P453S	H5Nx	- In silico predicted as a convergent or parallel evolution sites of H5Nx viruses.	[215]
NA	R292K	H7N9	- R292K substitution was found to promote drug resistance to oseltamivir in H7N9 viruses.	[266]
	H252Y		- H252Y increases the affinity of NA for oseltamivir, leading to increased antiviral susceptibility.	[267,268]
	H274Y E119A V116A I117V/T K150N D198N I222V/T/M S246N N247S N295S	H5N1 H1N1	- Reduce drug susceptibility in avian and human H1N1 and H5N1 viruses. - H274Y mutation results in enhanced resistance to oseltamivir. - E119A mutation increases resistance to zanamivir.	[239,267,268]
	I222R	H5N1 H1N1	- I222R mutation identified in clinical isolates receiving oseltamivir treatment, resulting in reduced susceptibility to all NA inhibitors (oseltamivir carboxylate, zanamivir, and peramivir).	[267]
	I222R H274Y	H5N1	- I222R + H274Y mutations enhance viral resistance to oseltamivir and zanamivir.	[267]
	I8T I/V16A V16I V33I N39S P45T K55R A58T K241R I243V N305T G318S P323S S364N G365E I380V V404I N430S/D G435S T441D	H5Nx	- In silico predicted as convergent or parallel evolution sites of H5Nx viruses.	[215]
M1	N30D T215A	H5N1	- An important determinant of H5N1 virus pathogenicity and lethality in mice.	[197,244]
	I43M	H5N1	- An important determinant of H5N1 AIVs in both avian and mammalian hosts.	[198]
	T137A A239T C269Y V280I S283N D340N	H5Nx	- In silico predicted as parallel evolved variations. - T137A is a human host marker.	[215]
M2	L26F/I V27A A30T/V S31N G34E L38F	HxNx	- Enhance adamantane resistance.	[75,244,267,269]
NS1	F55C	H5N1	- Stimulates polymerase activity and enhances viral replication.	[219,270]
	D120N	H5N1	- Possibly enhances viral replication or host adaptation.	[219]
	Δ263–277 D92E	H5N1	- Δ263–277 deletion together with D92E increase the virulence of H5N1 in chickens and mice. - D92E mutation contributes to regulation of type I interferon (IFN) levels.	[212,271]
	N200S G205R	H5N1	- Enhanced inhibition of type I IFN leads to increased virulence in ferrets. - N200S and G205R have been included as virulence markers for H5N1 viruses.	[212,261]
	F103L M106I	H5N1	- Increase inhibition of type I IFN antagonism and mediate interstitial pneumonia in mice. - Contribute to the host-adaptation ability of H5N1, H7N9, and H6N1 viruses.	[212,272]
	P42S	H5N1	- Regulation of IRF3 and IFN levels.	[200,212]
	Δ80–84	H5N1	- Role in the regulation of TNF-α levels.	[212,272,273]
	F138Y	H5N1	- Interacts with cellular PDZ proteins and Akt activation.	[212,274]
	S205N	H5Nx	- Decreases inhibition of type I IFN.	[215]
	G47S N48S R59H R67Q E70K T81I R88C V136A I137V D139N L185F D209N V209I L212F	H5Nx	- In silico predicted mutations as parallel evolved variations in H5Nx viruses.	[209,215]
	M/A14V A48T T/V115A	H5Nx	- In silico predicted mutations as parallel evolved variations in H5Nx viruses.	[209,215]
NS2	M/A14V A48T T/V115A		- In silico predicted mutations as parallel evolved variations in H5Nx viruses.	[209]

## Data Availability

The data presented in this study are available in the article.
